# Privacy–preserving dementia classification from EEG via hybrid–fusion EEGNetv4 and federated learning

**DOI:** 10.3389/fncom.2025.1617883

**Published:** 2025-08-18

**Authors:** Muhammad Umair, Muhammad Shahbaz Khan, Muhammad Hanif, Wad Ghaban, Ibtehal Nafea, Sultan Noman Qasem, Faisal Saeed

**Affiliations:** ^1^School of Engineering, University of Southern Queensland, Toowoomba, QLD, Australia; ^2^School of Computing, Engineering and the Built Environment, Edinburgh Napier University, Edinburgh, United Kingdom; ^3^Department of Informatics, School of Business, Örebro Universitet, Örebro, Sweden; ^4^Applied College, University of Tabuk, Tabuk, Saudi Arabia; ^5^College of Computer Science and Engineering, Taibah University, Medina, Saudi Arabia; ^6^Computer Science Department, College of Computer and Information Sciences, Imam Mohammad Ibn Saud Islamic University (IMSIU), Riyadh, Saudi Arabia; ^7^King Salman Center for Disability Research, Riyadh, Saudi Arabia; ^8^College of Computing, Birmingham City University, Birmingham, United Kingdom

**Keywords:** neurobehavior analysis, EEGNET, dementia, federated learning, deep learning, smart healthcare, hybrid-fusion, FedAvg

## Abstract

As global life expectancy rises, a growing proportion of the population is affected by dementia, particularly Alzheimer's disease (AD) and Frontotemporal dementia (FTD). Electroencephalography (EEG) based diagnosis presents a non-invasive, cost effective alternative for early detection, yet existing methods are challenged by data scarcity, inter-subject variability, and privacy concerns. This study proposes lightweight and privacy-preserving EEG classification framework combining deep learning and Federated Learning (FL). Five convolutional neural networks (EEGNetv1, EEGNetv4, EEGITNet, EEGInception, EEGInceptionERP) have been evaluated on resting-state EEG dataset comprising 88 subjects. EEG signals are preprocessed using band-pass (1–45 Hz) and notch filtering, followed by exponential standardization and 4-second windowing. EEGNetv4 outperformed among other EEG tailored models, and upon utilizing the hybrid fusion techniques it achieves 97.1% accuracy using only 1,609 parameters and less than 1 MB of memory, demonstrating high efficiency. Moreover, FL using FedAvg is implemented across five stratified clients, achieving 96.9% accuracy on the hybrid fused EEGNetV4 model while preserving data privacy. This work establishes a scalable, resource-efficient, and privacy-compliant framework for EEG-based dementia diagnosis, suitable for deployment in real-world clinical and edge-device settings.

## 1 Introduction

Over the past several decades, advances in medical science have steadily increased human life expectancy (Byles, 2024). As a result, the proportion of older adults in the population is growing worldwide (Safiri et al., 2023). This demographic shift brings new challenges for healthcare such as the cognitive abilities that change with the natural aging process (Arenaza-Urquijo et al., 2024). Aging brings higher risk of chronic health conditions, and it often produces gradual decline in cognitive functions such as memory, visuo-spatial processing, and executive control in the brain (Zandbagleh et al., 2024; Arenaza-Urquijo et al., 2024). In most cases, these mild changes affect the daily life of elderly people. However, in elderly adults (especially those over ninety years of age) cognitive impairment often exceeds normal aging expectations and instead signals an underlying neuro-degenerative disease (Zandbagleh et al., 2024). When these impairments begin to impact daily life specially due to memory issues, these conditions can be classified clinically as dementia (Zandbagleh et al., 2024). Patients with dementia lose short–term memory, struggle with language, and cannot plan or execute everyday tasks. According to World Health Organization (WHO) 57 million people had dementia in 2021 and every year 10 million cases arises (World Health Organization, 2025). In another report by Ministry of Health, Saudi Arabia, it has been mentioned that dementia is a growing health issue and Saudi Arabia is estimated to have a dementia prevalent of 5% for the age group beyond 65 which is an alarming number for a country (Ministry of Health, Kingdom of Saudi Arabia, 2024).

The two most common neuro-degenerative dementia are Alzheimer's Dementia (AD) and Frontotemporal Dementia (FTD) (Zandbagleh et al., 2024). AD accounts for the majority of dementia cases and arises from the accumulation of amyloid plaques and tau protein tangles in the hippocampus and cerebral cortex (DeTure and Dickson, 2019; Tönnies and Trushina, 2017). These abnormal deposits trigger neuron death and synaptic dysfunction. Patients first show impaired ability to form new memories (DeTure and Dickson, 2019; Tönnies and Trushina, 2017). They develop confusion, disorientation, and difficulty performing routine activities (DeTure and Dickson, 2019; Tönnies and Trushina, 2017). FTD targets the frontal and temporal lobes, the regions that govern behavior, personality, and language. It often stems from aberrant tau or TDP–43 protein inclusions. Early signs include changes in social conduct, loss of empathy, and progressive speech difficulty. As neurons degenerate, patients exhibit blunted emotion, compulsive behaviors, or aphasia, depending on which lobe suffers greatest injury (Bang et al., 2015; Boeve et al., 2022).

Traditionally, clinicians have diagnosed and monitored AD and FTD using a combination of clinical assessment, cognitive testing, and structural imaging (Dickerson et al., 2025). Standard tools like the mini mental state examination and the montreal cognitive assessment quantify deficits in memory and language functions (Wang et al., 2022). Similarly, magnetic resonance imaging and computed tomography reveal characteristic patterns medial temporal shrinkage in AD and FTD (Davatzikos et al., 2008). While these methods are being practiced to diagnose and detect the AD & FTD, however, they are costly, invasive, and may not be sensitive to early functional changes (Liss et al., 2021). However, Electroencephalography (EEG) offers a noninvasive method to record the brain's electrical activity in real time (Umair et al., 2025). For EEG recordings, clinicians place electrodes on the scalp to capture voltage fluctuations that reflect underlying neural oscillations (Lisgaras et al., 2025). For AD, EEG typically shows reduced power in the alpha band and increased slow wave activity (Umair et al., 2025). Whereas in FTD, slowing often concentrates over frontal leads (Boeve et al., 2022). Traditional EEG analysis relies on visual scoring and basic spectral measures (Lisgaras et al., 2025). Recent advances in Artificial Intelligence (AI) offer automated feature extraction and classification from EEG recordings enhancing early detection of AD and FTD.

Despite the promising results, AI–based EEG classification faces significant technical hurdles. Traditional Machine Learning (ML) methods require experts to handcraft features such as spectral power, temporal dynamics, and network connectivity and these engineered representations often fail to generalize across EEG datasets collected with different hardware, protocols, or patient populations (Azami et al., 2025). In contrast, Deep Learning (DL) architectures particularly Convolution Neural Networks (CNNs) automatically learn hierarchical spatial and temporal patterns directly from raw EEG time series and yields better accuracy in AD and FTD classification tasks (Umair et al., 2025). However, these end–to–end models typically include millions of trainable parameters and demand extensive floating–point operations. Training them requires powerful GPUs, while inference on portable EEG headsets or edge devices proves infeasible due to limited processing power and memory.

Moreover, the centralized data collection paradigm that underlies most DL & ML based EEG studies raises serious privacy and regulatory concerns. Aggregating sensitive EEG recordings on a single server conflicts with data–protection laws (e.g., GDPR) and endangers patient confidentiality (Natarajan and Shanthi, 2025). To address this, Federated Learning (FL) distributes model training across multiple clinical sites: each site trains a local model on its own data and shares only encrypted weight updates with a coordinating server (McMahan et al., 2017; Umair et al., 2024). This approach safeguards raw EEG signals, complies with privacy regulations, and improves scalability by parallelizing computation. Combining lightweight DL models with FL thus emerges as a compelling solution for real–time, privacy–preserving EEG diagnostics in clinical and edge–device settings.

This study addresses the dual challenges of data privacy and model compactness in EEG-based dementia classification by proposing a hybrid–fusion DL model and integrating it within FL framework. The main contributions are:

1. A comprehensive evaluation of five lightweight EEG architectures (EEGNetv1, EEGNetv4, EEGITNet, EEGInception, and EEGInceptionERP) on a resting–state EEG dataset. And proposed a hybrid–fusion based EEGNetv4 that combines global average and self-attention heads.
2. Deployment of the proposed hybrid–fusion EEGNetv4 model in FL setting across five simulated clinical clients, preserving patient privacy without sharing raw EEG. Along with detailed exploratory data analysis, preprocessing pipeline and hyperparameter tuning procedures that ensure suitability for real–time.

The remainder of the paper is organized as follows. Section 2 describes the related work and their discussion. the dataset and preprocessing techniques. Section 3 gives brief explanation about the utilized data preprocessing techniques, DL models and fusion technique along with its setup in FL environment. Section 4 presents experimental results and discussion. And, Section 5 concludes and outlines future directions.

## 2 Literature review

Recently, researchers have proposed DL solutions for classifying AD and FTD using EEG recordings. For example, a study by Jiang et al. (2025) used a publicly available EEG dataset (Miltiadous et al., 2023a) that includes resting-state, eyes–closed recordings from 36 AD patients, 23 FTD patients, and 29 cognitively normal controls. The study focused on analyzing functional connectivity through pairwise coherence values across different frequency bands. These coherence values served as connectivity fingerprints, revealing stronger global brain network connections in healthy controls, while both dementia groups exhibited disrupted connectivity patterns. Using these coherence matrices as images, the authors introduced a Coherence-CNN to classify the three groups (AD, FTD, and CN), achieving an accuracy of 94.3%.

Similarly, Zheng et al. (2025) used the same dataset (i.e., Miltiadous et al., 2023a) and applied time-frequency analysis to isolate key frequency bands. They then quantified pairwise functional connectivity using pearson correlation, mutual information, and the phase-lag index. The resulting connectivity matrices were used as features for a support vector machine (SVM), evaluated with leave-one-out cross-validation. Their results showed reduced theta-band connectivity in frontal brain regions and increased beta-band coupling in posterior regions for both AD and FTD. Additionally, only AD patients displayed a further reduction in theta-band connectivity in central brain regions. Their SVM classifier achieved 95% accuracy for AD vs. healthy controls and 86% accuracy for FTD vs. healthy controls. Lal et al. (2024) also worked with the same dataset (i.e., Miltiadous et al., 2023a) and employed various feature extraction techniques to distinguish AD and FTD from healthy controls. They used a 90% overlapping sliding window to segment the EEG signals, followed by Singular Value Decomposition (SVD) entropy for feature extraction. For classification, they applied K-Nearest Neighbors (KNN) algorithm. Their results showed accuracy of 91% for classifying AD vs. healthy controls, 93% for FTD vs. healthy controls, and 91% for distinguishing AD from FTD. Building on the same open resting–state EEG dataset (i.e., Miltiadous et al., 2023a), Rostamikia et al. (2024) extracted a broad set of frequency, time, complexity, and connectivity based features from the EEG recordings. After ranking each measure with t–tests and Mann–Whitney tests, they kept only the most informative channels and bands for dementia detection. SVM trained on these reduced feature sets, evaluated with ten–fold cross–validation, reached 93.5% accuracy for dementia vs. control and 87.8% for AD vs. FTD. Kassem and Kabbara (2024) used the same EEG repository to test whether a network oriented DL model could improve diagnostic accuracy. They converted each recording into a graph that represents functional links between electrodes, then fed those graphs to Graph Neural Network (GNN). In comparisons with standard classifiers (SVM, Random forest, KNN, Logistic regression, Naïve Bayes), the GNN provided the highest discrimination, cleanly separating AD from the combined set of FTD and healthy controls and likewise distinguishing FTD from healthy ones. They achieved an accuracy of 92.33%.

However, as mentioned in Section 1, that these DL and ML based solutions traditionally follow centralized method, where the dataset needs to be collected and gathered at one node, thus making them not suitable for practical use case because of data privacy concerns. Researchers have also proposed their studies where FL have been used as decentralized solution, such as Sahid et al. (2024) trained a CNN within FL framework. EEG data were split among clients under three scenarios. In each communication round every client performed several local epochs before sending weight updates to coordinating server, designed to reduce bandwidth usage while protecting raw data. With full client participation the FL system reached 84.75% accuracy (precision = 86%, recall = 85%, F1 score = 84%). Lakhan et al. (2024) propose FDCNN–AS, a federated deep–convolutional framework that trains across many hospitals while keeping patient data private. Unlike EEG–only efforts, their model fuses several modalities PET, SPECT, MRI, blood biomarkers, and cognitive questionnaires to capture a wider picture of neuro degeneration across age groups. Each clinic trains a local CNN and shares only model weights with a central server, meeting privacy requirements yet benefiting from larger pooled sample. Tested on the distributed dataset, FDCNN–AS reaches 99% accuracy in identifying AD. Similarly, Umair et al. (2025) tackle the same privacy and resource limits highlighted in dementia research, but test their solution on another EEG application major depressive disorder. They first benchmark standard models (Random forest, SVM, gradient boosting) alongside deep networks (transformers, auto–encoders). A transformer random forest ensemble outperformed at 99% accuracy when trained centrally. To remove the need for data pooling, they then adopt split learning (a recent advance concept in FL). With three independent clients this configuration still exceeds 95% accuracy proving that distributed and decentralized deep pipelines can match single–server baselines while meeting data–privacy rules with the federated strategies reported in dementia studies.

Moreover, real-time inference on edge devices demands models that are both fast and lightweight. Large DL networks, with millions of parameters and high computational load, introduce latency and slow response time that is an obstacle for hardware that offers limited memory and processing power. To overcome this bottleneck, researchers now focus on lightweight architectures. For example, Asif et al. (2024) proposed LiteFusionNet by running MobileNet and MobileNetV2 in parallel and concatenating their feature maps, so accuracy rises without adding heavy layers. The dual–backbone fusion keeps parameters lean and inference fast for real–time medical imaging. Gligorijević et al. (2018) introduce deepNF, a multimodal autoencoder that first learns modality–specific features from each interaction network, then merges them in a shared bottleneck layer to produce a single low–dimensional embedding. This fusion through a compact bottleneck captures complex relationships while sharply reducing feature size and model complexity. Liu and Mei (2023) achieve compactness by running an improved sparse PCA on each omics layer, turning high–dimensional profiles into terse similarity graphs that are later fused. Feeding this low–rank composite into a small DNN keeps parameters lean, curbs over–fitting, and still delivers accurate drug–response predictions.

Building on the gaps, this study introduces lightweight deep network that fuses complementary EEG features within the architecture, keeping parameters small while still separating AD, FTD, and healthy controls with high accuracy. The model trains under FL framework so each client retains its data, meeting privacy rules and enabling real–time deployment on resource–constrained edge devices.

## 3 Methods and materials

**Figure 1:**
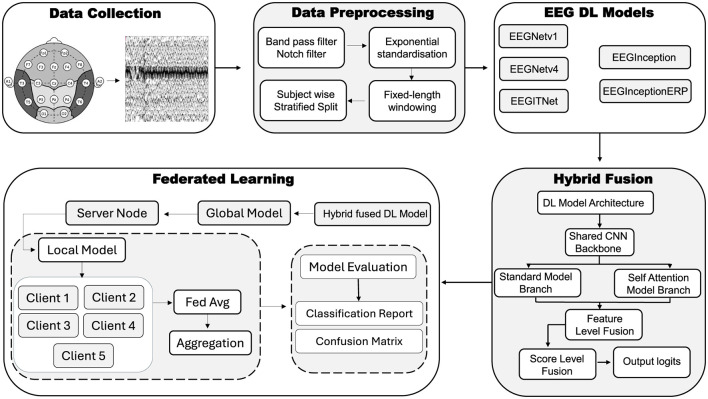
Overview of the utilized methodology using EEG signals.

This section briefly describes the dataset, the data–preprocessing pipeline, EEG–based DL models and their architectures, proposed hybrid–fusion technique and its integration, and the experiments conducted within FL environment. An overview of the utilized methodology has been depicted in Figure 1, that comprises of following important parts: Data collection, Data preprocessing, EEG DL Models, Hybrid Fusion and FL respectively.

### 3.1 Dataset

In this study, publicly available dataset (i.e., Miltiadous et al., 2023a), has been accessed. This dataset was initially used and introduced by Miltiadous et al. (2023b). This dataset was collected in resting–state, eyes–closed EEG corpus comprising 88 adult participants. The cohort consisted of 36 patients with AD, 23 patients with FTD and 29 cognitively normal (CN) controls. Cognitive status was quantified with the Mini–Mental State Examination (MMSE; range 0–30). EEG recordings were obtained with Nihon Kohden EEG–2100 clinical system using 19 scalp electrodes (i.e., Fp1, Fp2, F7, F3, Fz, F4, F8, T3, C3, Cz, C4, T4, T5, P3, Pz, P4, T6, O1, and O2) positioned according to the international 10–20 system. Signals were digitized at 500 Hz with hardware resolution of 10*V*/*mm*. An anterior-posterior bipolar montage and Cz–referential montage were recorded. Amplifier settings for sensitivity were kept to be 10*V*/*mm* and analog low–pass filter at 70 Hz. Thus, this dataset supplies balanced, high–quality resting–state EEG suitable for developing and evaluating DL algorithms for AD, FTD, and CN classification.

**Figure 2:**
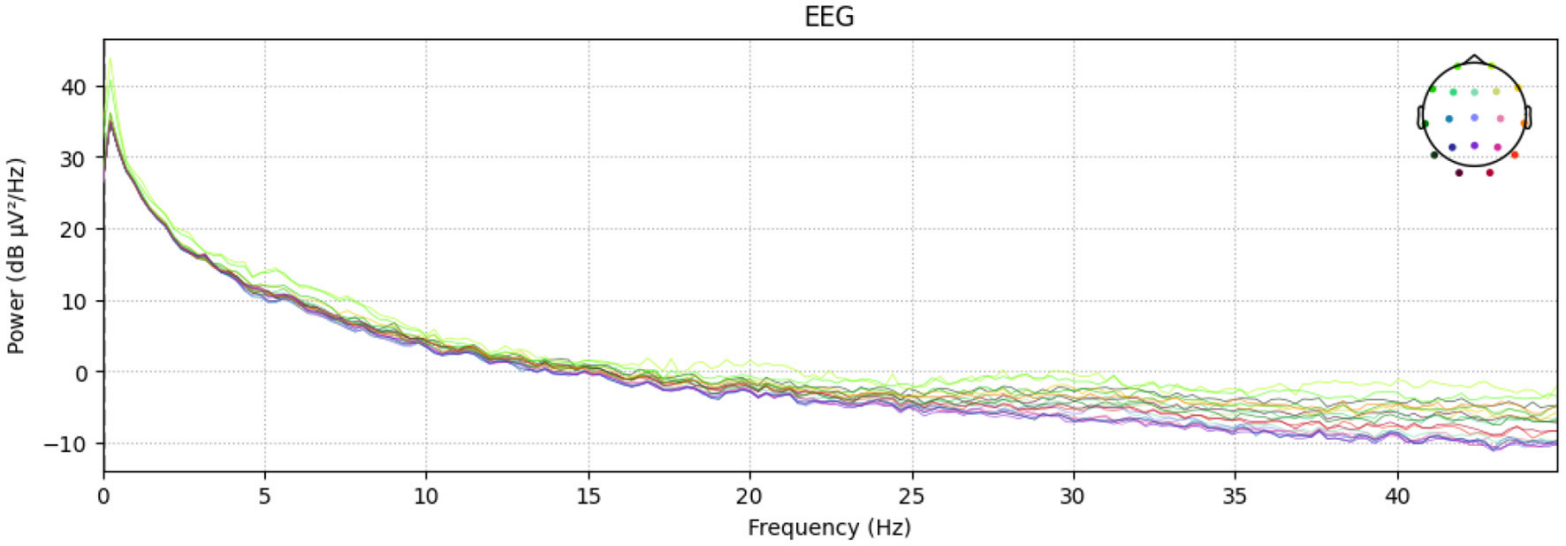
Channel–wise power–spectral density.

**Figure 3:**
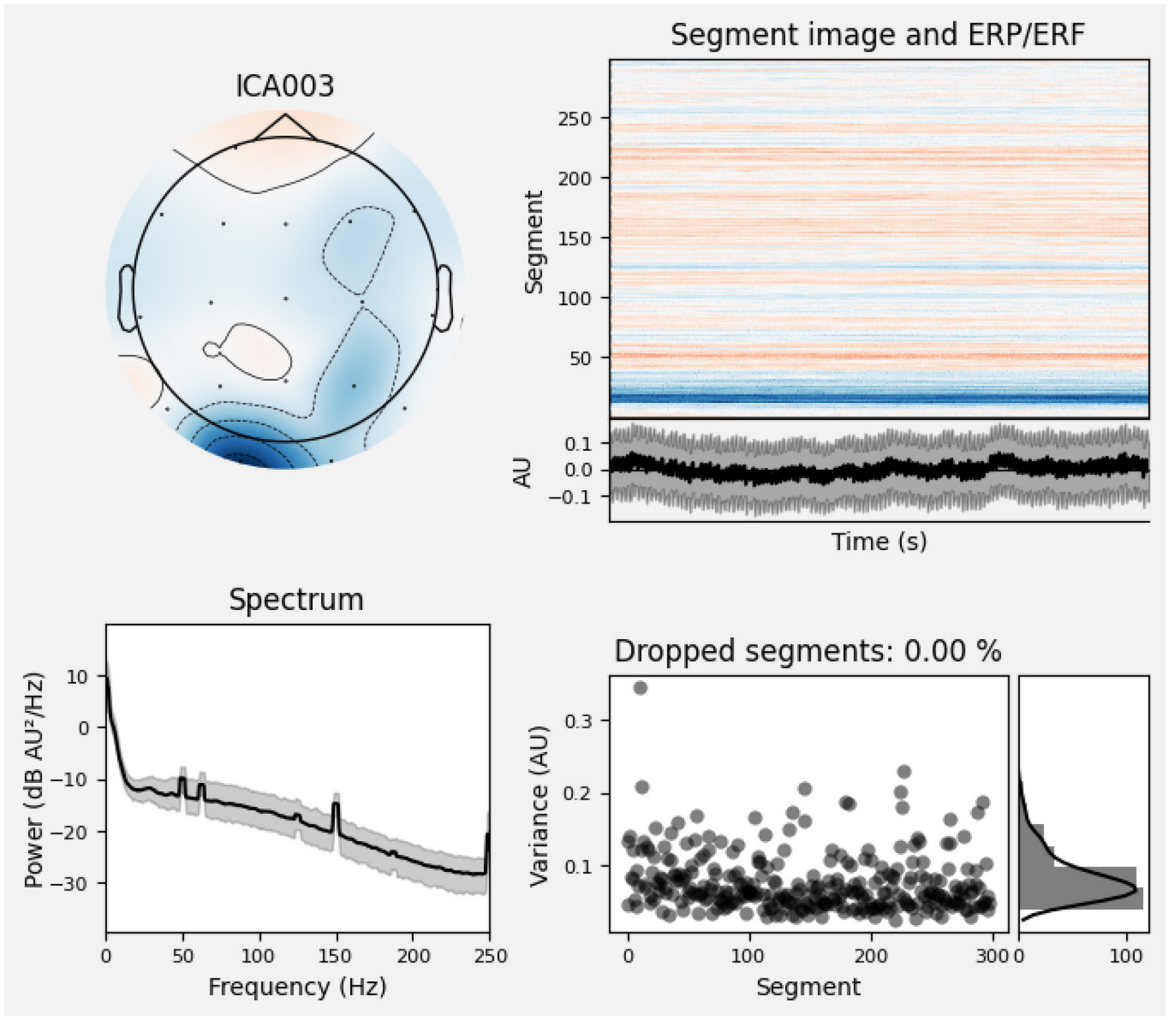
Diagnostic summary for ICA component.

Furthermore, Figure 2 depicts the power–spectral density of single pre–processed EEG recording (0–45 Hz), with each colored trace representing one of the 19 scalp electrodes. The plot shows the 1/f power decline along with a visible alpha peak (8–12 Hz) over posterior channels, while the absence of a 50/60 Hz spike confirms effective line–noise removal. This spectrum sample indicates clean, physiologically plausible data for subsequent analysis. And Figure 3 presents the diagnostic summary for ICA component. The scalp topography shows a strong frontal projection, while the segment image and overlaid average time course exhibit large slow deflections across epochs. The power spectrum is dominated by low–frequency (< 5 Hz) activity, and the variance scatter highlights several high-variance segments. These features identify this component that was being collected.

#### 3.1.1 Exploratory data analysis and visualization

**Figure 4:**
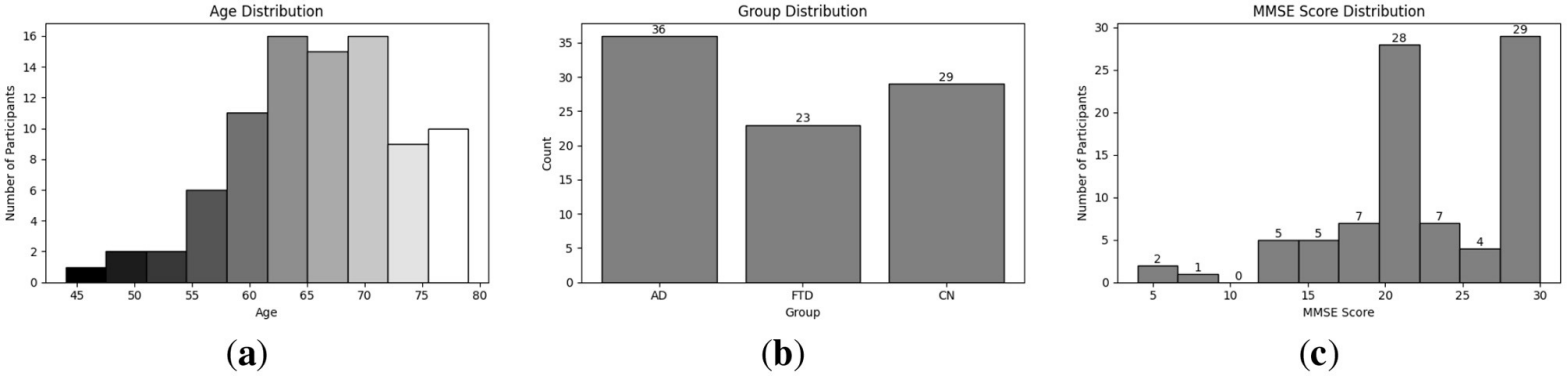
EDA on the utilized dataset; **(a)** Distribution of different age group in the utilized dataset; **(b)** Distribution of different disease group in the utilized dataset; **(c)** MMSE score distribution among participant.

**Figure 5:**
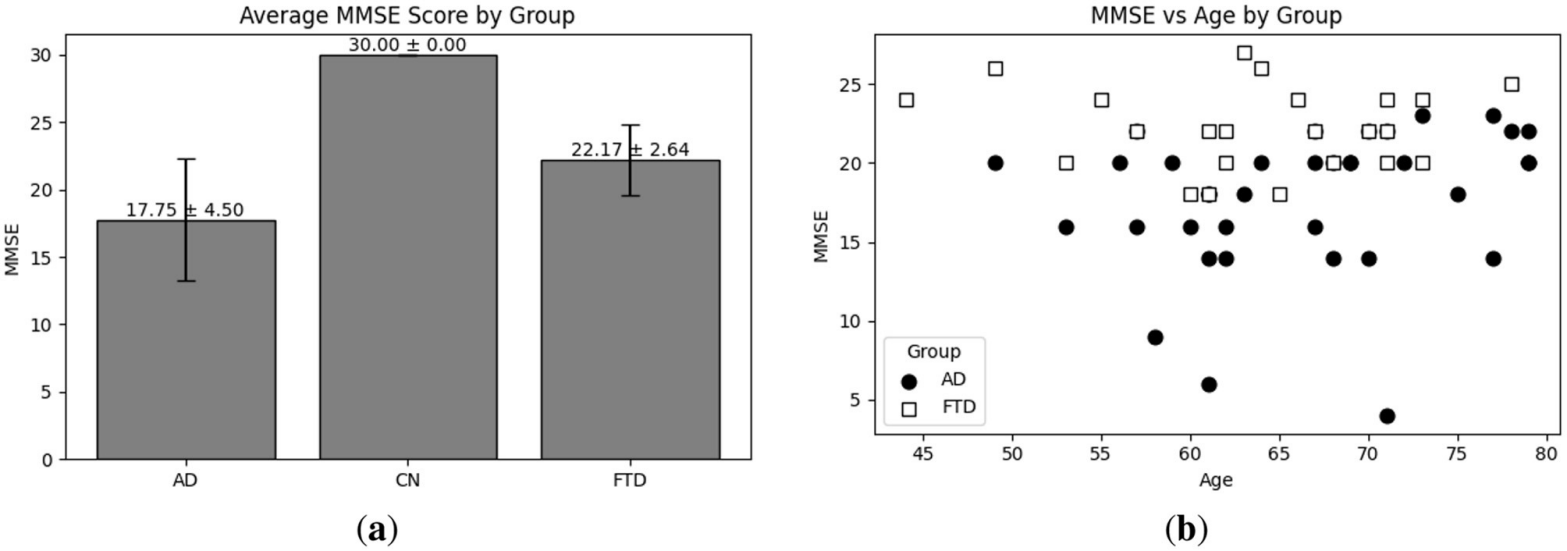
EDA on the utilized dataset; **(a)** Average MMSE score distribution with respect to each group; **(b)** MMSE score vs. age distribution with respect to each group (i.e., AD and FTD).

**Figure 6:**
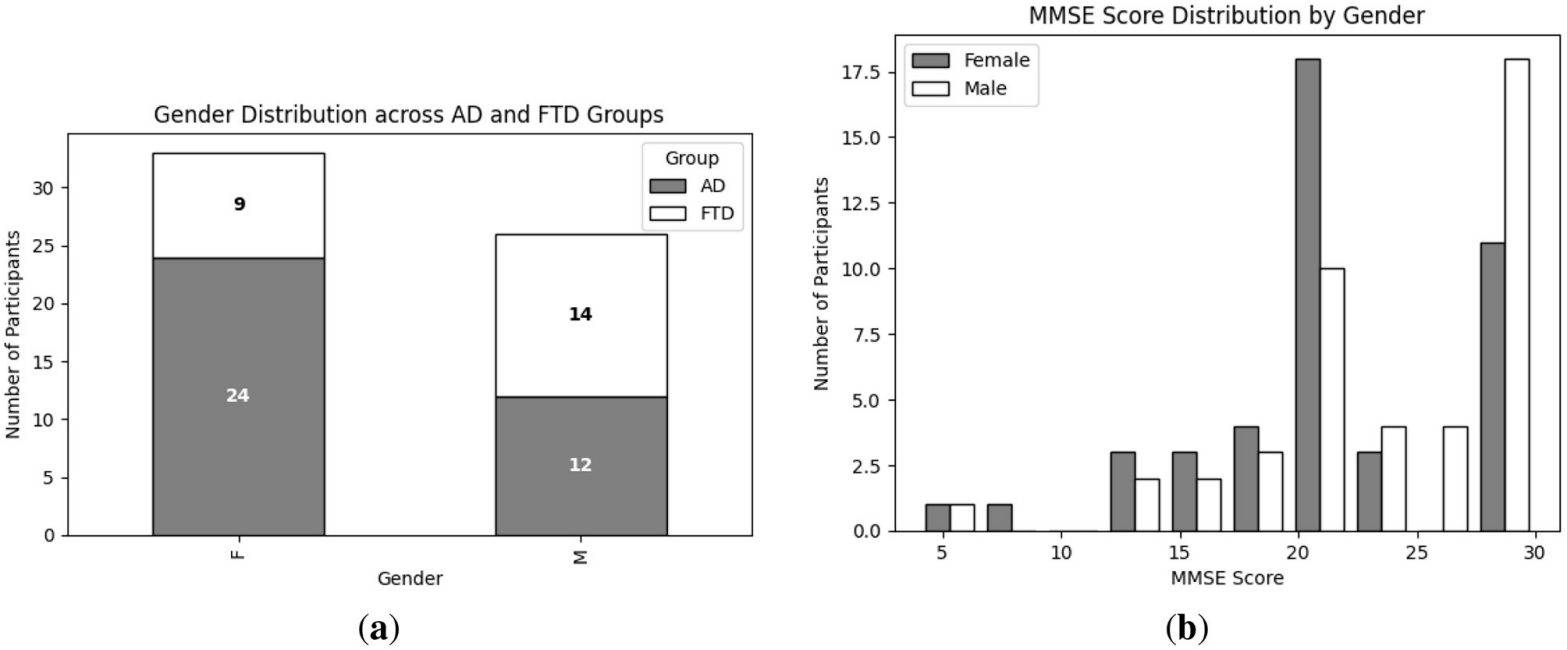
EDA on the utilized dataset; **(a)** Gender wise distribution of each group (i.e., AD and FTD); **(b)** MMSE score distribution of each group (i.e., male and female).

Exploratory Data Analysis (EDA) provides an essential overview of the dataset's demographic and clinical characteristics, guiding preprocessing and model selection. As shown in Figure 4a, participant ages span from 45 to 79 years, with the highest count in the 60–65 year. Figure 4b presents the diagnostic composition 36 AD, 23 FTD, and 29 CN subjects yielding a balanced dataset. Figure 4c illustrates MMSE scores ranging from 4 to 30, concentrated in the 20–23 and 28–30 intervals, while Figure 5a reports group mean ± standard deviation of 17.75 ± 4.50 (AD), 22.17 ± 2.64 (FTD), and 30.00 ± 0.00 (CN). As seen in Figure 5b, two AD participants over 70 years record the lowest MMSE values (4 and 6), whereas FTD scores remain within 18–28 across all ages. Figure 6a shows sex distributions of 24 female and 12 male in AD vs. 9 female and 14 male in FTD, and Figure 6b confirms nearly identical MMSE medians for males (23) and females (21) with overlapping inter quartile ranges. These analyses verify that the dataset is suitably diverse and free of extreme biases for training a robust DL classifier.

### 3.2 Data preprocessing

As shown in Figure 1, several techniques are implemented for data preprocessing on EEG signals. Techniques like filtering of signals (i.e., band pass filter and Notch filter), exponential standardization, fixed-length windowing and then subject wise stratified split techniques have been utilized; these are discussed below.

#### 3.2.1 Band pass and notch filter

A band–pass filter is a signal processing tool that admits only those frequency components within a specified range, rejecting both lower and higher frequencies (Christiano and Fitzgerald, 2003). In this study, raw EEG recordings first pass through a 1–45 Hz finite–impulse–response (FIR) band–pass filter. This step removes slow baseline drifts below 1 Hz often caused by sweating or movement—and attenuates high–frequency noise above 45 Hz, such as muscle artifact, while preserving the canonical EEG rhythms (delta through low–gamma) that carry markers of cognitive state. By focusing on this frequency band, the analysis emphasizes the alpha peak (8–12 Hz) changes associated with AD and FTD.

Whereas, a notch filter attenuates narrow frequency band around a known interference frequency, and a narrow band notch filter eliminates residual noise interference (Alhammadi and Mahmoud, 2016; Schirrmeister et al., 2017). In this study's clinical recordings, such interference can obscure subtle spectral features, so its removal ensures that subsequent normalization and feature extraction operate on physiologically meaningful signal components rather than electrical artifacts.

#### 3.2.2 Exponential standardization

Exponential standardization computes per-channel normalization by continuously updating the mean and variance with exponential decay. Exponential moving standardization is particularly well suited for EEG signals, which exhibit non stationary drifts and abrupt amplitude changes. In this study, python based library braindecode has been utilized in order to apply exponential standardization on EEG signal (Braindecode Developers, 2022). It computes the exponential moving mean *m*_*t*_ at time *t* and *K* is the factor new as given in Equation 1.


(1) $$\begin{array}{l} m_{t}=K*mean\left(X_{t}\right)+\left(1-K\right)*m_{\left(t-1\right)} \end{array}$$

And then, compute exponential moving variance *v*_*t*_ at time *t* as given in Equation 2.


(2) $$\begin{array}{l} v_{t}=K*{\left(m_{t}-X_{t}\right)}^{2}+\left(1-K\right)*v_{\left(t-1\right)} \end{array}$$

Thus, standardize the data point *X*_*t*_ at time *t* is given in Equation 3.


(3) $$\begin{array}{l} X_{t}^{\prime }=\frac{\left(X_{t}-m_{t}\right)}{max(\sqrt{\left(->v_{t}\right)},eps)} \end{array}$$

#### 3.2.3 Fixed length windowing

Fixed–length windowing takes chunks of each continuous EEG recording into short, uniform segments so that the model sees manageable, consistent snapshots instead of one long trace. By using 4–second windows with a 50% overlap (i.e., a new window starts every 2 seconds), the process both boosts the number of training examples and captures gradual changes in brain activity. In this work, fixed window length function generates these overlapping 4 second slices automatically, providing a varying set of inputs for the classifier.

#### 3.2.4 Subject wise stratified split

A subject–wise stratified split assigns entire recordings (and their derived windows) to training, validation and test sets while preserving the original class proportions. Utilizing scikit–learn's with the stratify option on each subject's label, 80% of participants (23 AD, 14 FTD, 19 CN) populate the training set, 20% of the remainder form the validation set (6 AD, 4 FTD, 4 CN), and the final 20% constitute the test set (7 AD, 5 FTD, 6 CN). Table 1 details the number of subjects and resulting 4 second windows in each partition. This approach prevents windows from the same individual appearing in multiple splits, thereby avoiding data leakage and ensuring that each diagnostic group remains proportionally represented during model development.

**Table 1:** Number of samples among train, valid and test set.

**Classes**	**Train**		**Valid**		**Test**	
	**Samples**	**Windows**	**Samples**	**Windows**	**Samples**	**Windows**
AD	23	9,072	6	2,516	7	2,837
FTD	14	5,522	4	1,677	5	2,026
CN	19	7,494	4	1,677	6	2,431

### 3.3 EEG DL models

Five CNN architectures utilized by the braindecode framework are evaluated: EEGNetv1 (Lawhern et al., 2018), EEGNetv4 (Lawhern et al., 2018), EEGITNet (Salami et al., 2022), EEGInception (Santamaría-Vázquez et al., 2020), and EEGInceptionERP (Santamaría-Vázquez et al., 2020). All models are given an inputs shape of 19 channels × 512 samples and they provide output in three class scores. Table 2 summarizes each architecture's key layers. In the table, Params denotes the total number of learnable parameters; MAdds is the memory (in MB) of all multiply–add operations [A multiply–add (multi-add) is the basic arithmetic step in every convolution i.e., Two floating-point operations (1 multiply + 1 add)] for one forward pass; In is the input tensor size; F/B is the feature–map memory during forward/backward passes; Wts is the memory for the model's weights; and Total is the sum of input, feature–map and weight memory. The layers column uses the following abbreviations: Temp (temporal convolution), Spat (spatial convolution), Sep (depthwise–separable convolution), DW (depthwise convolution), Pool (pooling), DO (dropout), 1 × 1 (pointwise convolution), TConv (depthwise temporal-conv block + activation + dropout), Incep (Inception block with parallel temporal kernels), ELU (exponential linear unit activation), FC (fully–connected layer), and Incep (ERP) (Inception block tuned for event–related potentials).

**Table 2:** Architectural summary and resource requirements of the EEG deep-learning models.

**Model**	**Layers**	**Params**	**MAdds**	**In**	**F/B**	**Wts**	**Total**
			**(MB)**	**(MB)**	**(MB)**	**(MB)**	**(MB)**
EEGNetv1	Temp, Spat, Sep, Pool, DO, 1 × 1	2,539	2.83	0.04	0.72	0.01	0.77
EEGNetv4	Temp, DW, Sep, Pool, DO, 1 × 1	2,179	5.21	0.04	1.43	0.01	1.48
EEGITNet	Incep, 2 × TConv, Pool, DO, FC	4,833	6.79	0.04	2.58	0.02	2.64
EEGInception	4 × Incep, Pool, ELU, FC	22,447	10.95	0.04	4.19	0.06	4.30
EEGInceptionERP	4 × Incep(ERP), Pool, ELU, FC	22,447	10.95	0.04	4.19	0.06	4.30

#### 3.3.1 EEGNetv1 model

EEGNetv1 begins with temporal convolution followed by spatial convolution, then applies depthwise-separable convolutions interleaved with pooling and dropout, and ends in 1 × 1 convolutional head. As given in Table 2, this model contains 2539 learnable parameters, requires 2.83 MB of mult-add operations, occupies 0.04 MB for the input buffer, 0.72 MB for intermediate forward/backward feature maps, 0.01 MB for parameters, and an estimated total memory footprint of 0.77 MB.

#### 3.3.2 EEGNetv4 model

EEGNetv4 (as given in Table 2) refines the original by using constrained depthwise spatial convolution and factorized separable convolutions, with average-pooling and dropout after each block. Its classification head is again 1 × 1 convolution. This variant has 2179 parameters, 5.21 MB of mult-add cost, 0.04 MB input size, 1.43 MB for feature maps, 0.01 MB for weights, and total estimated size of 1.48 MB.

#### 3.3.3 EEGITNet model

EEGITNet starts with Inception block (parallel temporal filters), continues with two depthwise temporal-conv blocks and pooling, and finishes with a fully-connected layer with softmax activation function. As given in Table 2, it contains 4,833 parameters, 6.79 MB of mult-adds, 0.04 MB input buffer, 2.58 MB of feature-map memory, 0.02 MB for parameters, and totals 2.64 MB.

#### 3.3.4 EEGInception model

EEGInception stacks four Inception blocks each with multiple temporal-kernel branches—interleaved with average-pooling and ELU activations, then flattens to dense layer. As given in Table 2, this deep configuration model has 22,447 parameters, 10.95 MB of mult-adds, 0.04 MB input size, 4.19 MB for F/B maps, 0.06 MB of weights, and 4.30 MB.

#### 3.3.5 EEGInceptionERP model

EEGInceptionERP mirrors EEGInception's four-block Inception stack but uses ERP-optimized branch sizes. As given in Table 2, this model contains parameter count (22,447), mult-adds (10.95 MB), input footprint (0.04 MB), feature-map memory (4.19 MB), parameter memory (0.06 MB), and total size (4.30 MB).

### 3.4 Proposed hybrid fusion model

Proposed hybrid-fusion combines the strengths of two complementary processing paths one that captures overall signal patterns and another that focuses on how different time points relate to produce more reliable EEG classifications. For this study, EEGNetv4 outperformed as the results are later discussed in Section 4. And as illustrated in Figure 1, the network first applies a compact convolutional backbone to every input window, extracting basic temporal and spatial features through a series of small filters, pooling steps and dropout layers. After this shared feature extractor, the model splits into two branches. The standard branch averages each feature map over time and feeds the result into simple classifier, effectively summarizing during the window. The attention branch treats each time step as a token in a sequence, applies lightweight self-attention mechanism to let the model learn which moments are most informative, then also pools and classifies.

To bring these layers together, the outputs of both classifiers and a third fusion classifier are averaged into final logits as given in Equation 4.


(4) $$\begin{array}{l} {\text{z}}_{\text{final}}=\frac{{\text{z}}_{A}+{\text{z}}_{B}+{\text{z}}_{F}}{3} \end{array}$$

where **z**_*A*_, **z**_*B*_ and **z**_*F*_ are the prediction scores from the standard branch, the attention branch, and the fusion head, respectively. This hybrid approach offers several practical benefits. By combining two different perspectives, it is more robust to noise or unexpected signal changes in any single branch. Averaging the three classifiers helps smooth out mistakes, much like ensembling multiple models, without greatly increasing the total number of parameters. In fact, the proposed hybrid-fusion network requires only 1,609 parameters and occupies less than 1 MB of memory (0.04 MB for the input, 0.90 MB for intermediate feature maps and 0.01 MB for weights).

#### 3.4.1 Shared CNN backbone

The convolutional backbone applies series of small temporal and spatial filters to each 4s EEG window, isolating fundamental oscillatory patterns (e.g., alpha, beta bands) and electrode co-variation. Two rounds of pooling and dropout progressively reduce data dimensionality and guard against overfitting, while the final separable convolution efficiently captures more complex cross-channel dynamics without large increase in parameter count. This shared feature extractor serves as powerful common foundation for both downstream branches.

#### 3.4.2 Branch A: global feature head

Branch A pools each feature map over the entire time axis, condensing the tensor to fixed-length vector that represents the average power in each learned filter. A single dense layer then translates these summary statistics into class scores. This pathway excels at capturing broad, changes in brain rhythm amplitudes that are characteristic of different dementia types.

#### 3.4.3 Branch B: attention-enhanced head

Branch B reshapes the backbone output so that each time point becomes a token in short sequence. A lightweight multi-head self-attention layer learns to weigh these tokens against each other, highlighting critical moments such as transient bursts or slow drifts that may carry diagnostic information. After pooling, the resulting feature vector is classified in the same way as Branch A but with a focus on temporal relationships rather than average power.

#### 3.4.4 Fusion strategies

Two fusion steps unify the complementary information from the two branches. Feature–level fusion concatenates the two per-branch vectors into a single representation, which is then passed through an additional classifier to yield logits **z**_*F*_. Score–level fusion averages the three logits **z**_*A*_, **z**_*B*_, and **z**_*F*_into the final prediction according to Equation 4. This dual fusion both enriches the feature space and smooths out individual errors.

Despite its multi-branch design, the proposed hybrid-fusion model remains compact and fast: only 1,609 parameters in total, under 1 MB of memory footprint, and negligible additional inference overhead compared to a single EEGNetv4. The ensemble like fusion reduces variance and improves generalization, making the model well suited for real-time or resource–constrained applications.

### 3.5 Federated learning

**Figure 7:**
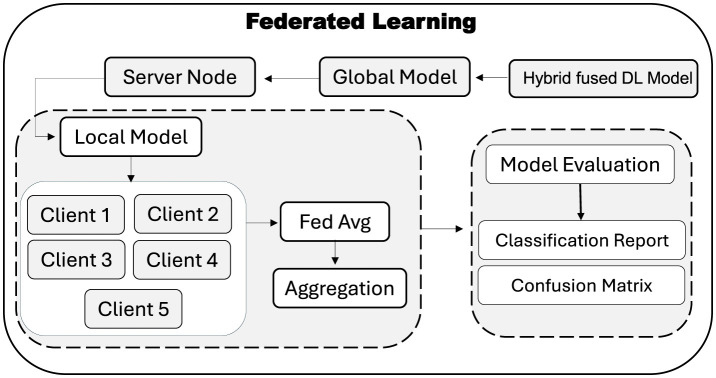
Utilized FL approach for the decentralized training.

Traditional centralized training of DL requires aggregating all data in one place, which raises serious privacy and security concerns for sensitive EEG recordings. To address this, FL approach is adopted for the proposed hybrid–fusion EEGNetv4 model. As shown in Figure 7, the global model is first initialized on a central server and then distributed to multiple client sites, each of which retains its own private data.

At each FL round, the server broadcasts the current global weights to every client. Each client trains the model locally on its own EEG windows for fixed number of epochs and returns only its updated weights. The server then aggregates these updates using the Federated Averaging (FedAvg) rule, which computes the new global weights as the average of all client-side weights, as given in Equation 5:


(5) $$\begin{array}{l} {\text{w}}_{\text{global}}=\frac{1}{N}\overset{N}{\underset{i=1}{\sum }}w_{i} \end{array}$$

Here, *w*_*i*_ denotes the weight vector from client *i* and *N* is the total number of participating clients. This aggregated model is then redistributed for the next round of local training. After convergence, the final global model resides on the server and can be evaluated on a held–out test set to produce a classification report and confusion matrix, without ever compromising participant privacy. FL thus enables collaborative model improvement across multiple institutions while ensuring that raw EEG data remains private.

#### 3.5.1 Client partitioning

In FL, each client represents a data-holding site that trains the model locally on its own subset of EEG windows. This decentralization preserves privacy raw signals never leave the client and naturally reflects real world scenarios where data are distributed across hospitals or research centers. Moreover, treating each site as client enables the global model to learn from heterogeneous data distributions, improving generalization.

#### 3.5.2 Participating clients and data distribution

Five clients participate in training, each receiving a stratified subset of the fixed length windows so that class proportions remain consistent across clients. Table 3 reports the number of windows per class (AD, FTD, and CN) for each client in the training, validation and test splits.

**Table 3:** Number of windows per class for each client across splits.

**Client**	**Train**			**Valid**			**Test**		
	**AD**	**FTD**	**CN**	**AD**	**FTD**	**CN**	**AD**	**FTD**	**CN**
Client 1	1,820	1,013	1,586	520	319	336	598	343	519
Client 2	1,820	1,012	1,586	520	319	336	598	343	519
Client 3	1,819	1,012	1,586	520	318	336	597	343	519
Client 4	1,819	1,012	1,586	520	318	335	597	342	519
Client 5	1,819	1,012	1,586	520	318	335	597	342	518

This stratified technique ensures that each client trains on a similar mix of AD, FTD and CN windows, preventing any single client from dominating the global update with a skewed class distribution. During each round, all five clients train the proposed hybrid-fusion EEGNetv4 on their local windows and send only weight updates to the server, which aggregates them via FedAvg (Equation 5) before redistributing the new global model.

## 4 Results and discussions

This section briefly discuss about the obtained results from the conducted experiments that then presented and analyzed. Model performance is measured on the test set that is given in Table 1, focusing on classification of AD, FTD, and CN for EEG set. Standard evaluation metrics Precision, Recall, F1–score and Accuracy are computed from the confusion matrix and the classification report. Each metric is defined below, along with its mathematical formulation. Precision quantifies the proportion of correct positive predictions and mathematically its given in Equation 6. Recall (as given in Equation 7) measures the proportion of actual positives that are correctly identified. The F1–score balances precision and recall via their harmonic mean, as represented in Equation 8. And accuracy (as given in Equation 9) reflects the overall fraction of correct predictions across all classes. Here in these equations, True Positive (TP) defines as number of windows correctly predicted as the given class. False Positive (FP) defines as number of windows incorrectly predicted as the given class. False Negative (FN) defines as number of windows of the given class incorrectly predicted as another class. And, True Negative (TN) defines as the number of windows neither belonging to nor predicted as the given class.


(6) $$\begin{array}{l} \text{Precision}=\frac{\text{TP}}{\text{TP}+\text{FP}} \end{array}$$

(7) $$\begin{array}{l} \text{Recall}=\frac{\text{TP}}{\text{TP}+\text{FN}} \end{array}$$

(8) $$\begin{array}{l} \text{F1-score}=2\times \frac{\text{Precision}\times \text{Recall}}{\text{Precision}+\text{Recall}} \end{array}$$

(9) $$\begin{array}{l} \text{Accuracy}=\frac{\text{TP}+\text{TN}}{\text{TP}+\text{TN}+\text{FP}+\text{FN}} \end{array}$$

In addition to the above metrics, balanced accuracy is computed to account for classes by averaging the recall (TP rate) across all classes. For *K* classes, it is defined in Equation 10.


(10) $$\begin{array}{l} \text{Balanced accuracy}=\frac{1}{K}\overset{K}{\underset{k=1}{\sum }}\frac{\text{T}{\text{P}}_{k}}{\text{T}{\text{P}}_{k}+\text{F}{\text{N}}_{k}} \end{array}$$

where TP_*k*_ and FN_*k*_ denote the true positives and false negatives for class *k*, respectively. This metric ensures that each class contributes equally to the overall score, regardless of its support.

### 4.1 Hyperparameter tuning

Grid search is employed to tune all critical hyperparameter of the utilized DL models by systematically evaluating each combination on the validation split and selecting the configuration that maximizes balanced accuracy. For the convolutional backbone, the number of temporal filters *F*_1_ is tested at 8 and 16, the depth multiplier *D* at 1 and 2, and the second-stage filter count *F*_2_ at 16 and 32. Temporal kernel lengths of 32 and 64 samples, dropout probabilities of 0.25 and 0.5, and attention-head counts of 2 and 4 are also explored. On the optimization side, learning rates of 10^−3^ and 10^−4^ under the Adam optimizer, batch sizes of 32 and 64, and total epochs of 50. Furthermore, an analogous grid search determines the FL parameters. The number of communication rounds *R* is tested at 20, with local training epochs per client ℓ of 10; client learning rates of 10^−3^ and 10^−4^; and federated batch sizes of 32.

### 4.2 EEG DL models results

#### 4.2.1 EEGNetv1

**Figure 8:**
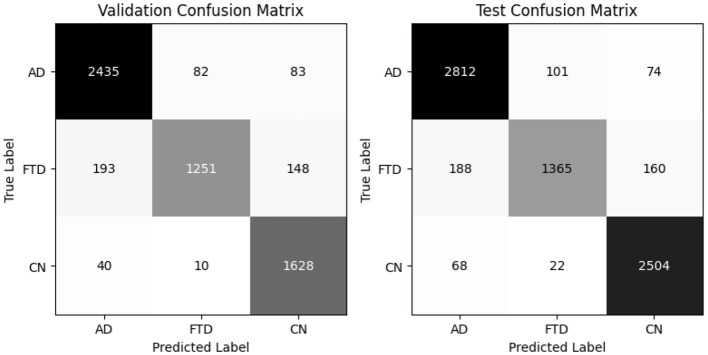
EEGNetv1 confusion matrix for valid and test set.

Table 4 reports the validation and test classification metrics for EEGNetv1. On the validation set, EEGNetv1 achieves overall accuracy of 91% and balanced accuracy of 89.80%, while on the test set it reaches 92% accuracy and 90.1% balanced accuracy. Class–level performance is strongest on the CN class (recall 0.97–0.97) and weakest on FTD (recall 0.79–0.80). The corresponding confusion matrices (as shown in Figure 8) reveal that most misclassifications occur between FTD classes.

**Table 4:** EEGNetv1: validation and test classification metrics.

	**Validation**				**Test**			
**Class**	**Precision**	**Recall**	**F1-score**	**Samples**	**Precision**	**Recall**	**F1-score**	**Samples**
AD	0.91	0.94	0.92	2,600	0.92	0.94	0.93	2987
FTD	0.93	0.79	0.85	1,592	0.92	0.80	0.85	1713
CN	0.88	0.97	0.92	1,678	0.91	0.97	0.94	2594
Accuracy	91%			5,870	92%			7,294
Balanced Acc.	89.80%				90.10%			

#### 4.2.2 EEGNetv4

**Figure 9:**
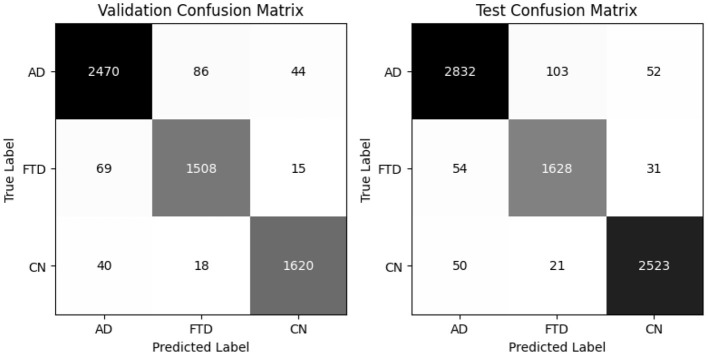
EEGNetv4 confusion matrix for valid and test set.

EEGNetv4 substantially improves (Table 5), reaching 95% accuracy and 95.40% balanced accuracy on validation, and 96% accuracy with 95.7% balanced accuracy on the test set. Precision and recall exceed 0.93 for all classes, demonstrating robust discrimination. Confusion matrices as shown in Figure 9 confirm misclassified samples.

**Table 5:** EEGNetv4: validation and test classification metrics.

	**Validation**				**Test**			
**Class**	**Precision**	**Recall**	**F1-score**	**Support**	**Precision**	**Recall**	**F1-score**	**Support**
AD	0.96	0.95	0.95	2,600	0.96	0.95	0.96	2,987
FTD	0.94	0.95	0.94	1,592	0.93	0.95	0.94	1,713
CN	0.96	0.97	0.97	1,678	0.97	0.97	0.97	2,594
Accuracy	95%			5,870	96%			7,294
Balanced Acc.	95.4%				95.7%			

#### 4.2.3 EEGITNet

**Figure 10:**
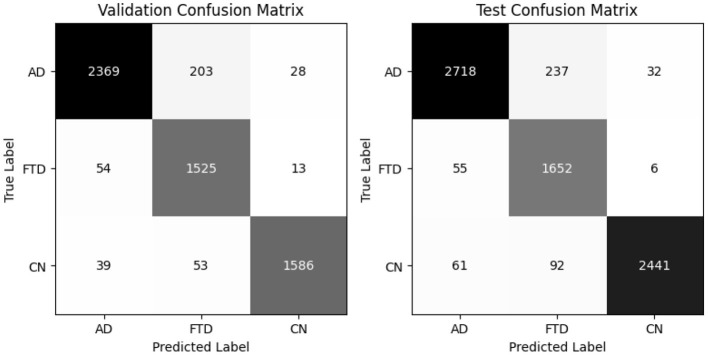
EEGITNet confusion matrix for valid and test set.

EEGITNet delivers 93% accuracy and 93.8% balanced accuracy on validation, and the same balanced accuracy of 93.8% with 93% test accuracy (Table 6). It shows better recall on FTD (0.96–0.96) but slightly lower precision for that class. The confusion matrices (as shown in Figure 10) indicate most FTD misclassifications fall into AD.

**Table 6:** EEGITNet: validation and test classification metrics.

	**Validation**				**Test**			
**Class**	**Precision**	**Recall**	**F1-score**	**Support**	**Precision**	**Recall**	**F1-score**	**Support**
AD	0.96	0.91	0.94	2,600	0.96	0.91	0.93	2,987
FTD	0.86	0.96	0.90	1,592	0.83	0.96	0.89	1,713
CN	0.97	0.95	0.96	1,678	0.98	0.94	0.96	2,594
Accuracy	93%			5,870	93%			7,294
Balanced Acc.	93.8%				93.8%			

#### 4.2.4 EEGInception

**Figure 11:**
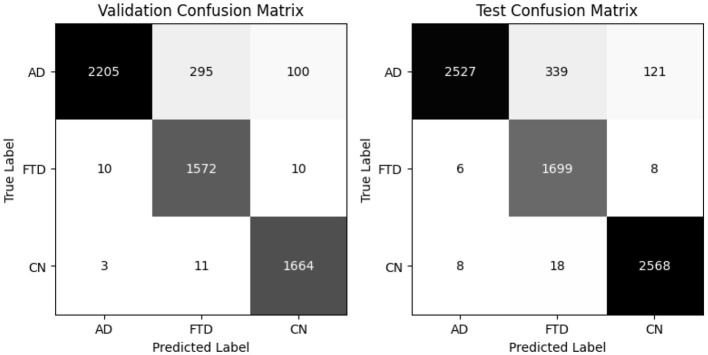
EEGInception confusion matrix for valid and test set.

EEGInception achieves 93% accuracy on both validation and test sets, with balanced accuracies of 94.2% and 94.3%, respectively (Table 7). It outperforme for CN class with recall (0.99) but shows moderate recall on AD (0.85). Confusion matrix as shown in Figure 11 confirm that AD windows are most often confused with FTD.

**Table 7:** EEGInception: validation and test classification metrics.

	**Validation**				**Test**			
**Class**	**Precision**	**Recall**	**F1-score**	**Support**	**Precision**	**Recall**	**F1-score**	**Support**
AD	0.99	0.85	0.92	2,600	0.99	0.85	0.91	2,987
FTD	0.84	0.99	0.91	1,592	0.83	0.99	0.90	1,713
CN	0.94	0.99	0.96	1,678	0.95	0.99	0.97	2,594
Accuracy	93%			5,870	93%			7,294
Balanced Acc.	94.2%				94.3%			

#### 4.2.5 EEGInceptionERP

**Figure 12:**
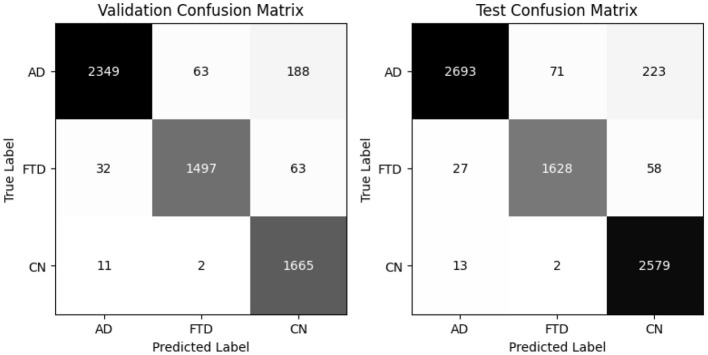
EEGInceptionERP confusion matrix for valid and test set.

EEGInceptionERP attains 94% validation accuracy (94.5% balanced) and 95% test accuracy (94.9% balanced accuracy) as shown in Table 8. It combines better recall on AD and FTD with high precision. Confusion matrices in as shown in Figure 12 show minimal misclassifications.

**Table 8:** EEGInceptionERP: validation and test classification metrics.

	**Validation**				**Test**			
**Class**	**Precision**	**Recall**	**F1-score**	**Support**	**Precision**	**Recall**	**F1-score**	**Support**
AD	0.98	0.90	0.94	2,600	0.99	0.90	0.94	2,987
FTD	0.96	0.94	0.95	1,592	0.96	0.95	0.95	1,713
CN	0.87	0.99	0.93	1,678	0.90	0.99	0.95	2,594
Accuracy	94%			5,870	95%			7,294
Balanced Acc.	94.5%				94.9%			

Thus, EEGNetv4 deliver the highest accuracy and balanced accuracy, while the Inception–based architectures excel in recall for specific classes. These detailed results guide the selection of the most suitable model for EEG–based dementia classification. And it is because of this reason only this model was chosen for the proposed hybrid-fusion technique.

### 4.3 Hybrid fusion with EEGNetv4

**Figure 13:**
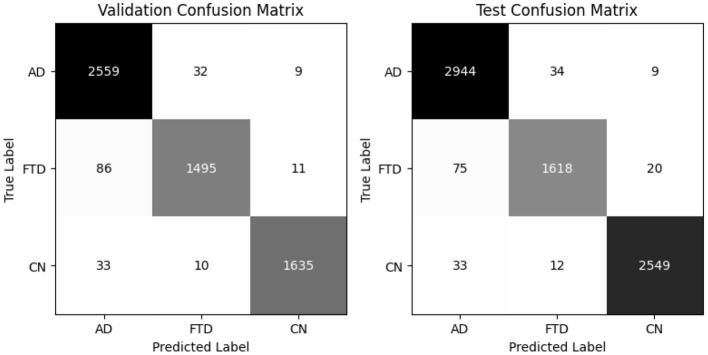
Proposed Hybrid-fused EEGNetv4 confusion matrix for valid and test set.

The hybrid-fusion EEGNetv4 model contains 1,609 trainable parameters and occupies 0.04 MB for the input buffer, 0.90 MB for intermediate feature maps during forward/backward passes, 0.01 MB for the parameter tensors, and has an estimated total memory footprint of 0.94 MB per training iteration. Table 9 summarizes its classification performance on the validation and test sets, and Figure 13 shows the corresponding confusion matrices.

**Table 9:** Hybrid-fusion EEGNetv4 results including Cohen's kappa and MCC.

	**Validation**				**Test**			
**Class**	**Precision**	**Recall**	**F1-score**	**Support**	**Precision**	**Recall**	**F1-score**	**Support**
AD	0.96	0.98	0.97	2,600	0.96	0.99	0.97	2,987
FTD	0.97	0.94	0.96	1,592	0.97	0.94	0.96	1,713
CN	0.99	0.97	0.98	1,678	0.99	0.98	0.99	2,594
Accuracy	97%			5,870	97%			7,294
Balanced Acc.	96.6%				97.1%			
Cohen's kappa	0.952				0.961			
MCC	0.953				0.961			

Beyond the usual precision & recall statistics, two reliability indices are reported i.e., Cohen's kappa and Matthews correlation coefficient (MCC). Cohen's kappa measures the agreement between predicted and true labels while correcting for chance. MCC provides a single balanced correlation score that remains informative even when the class distribution is skewed. On the validation set kappa = 0.952 and MCC = 0.953; on the test set kappa = 0.961 and MCC = 0.961. Both scores confirm that the hybrid-fusion network maintains best performance with ground truth across all three classes.

On the validation set, the model attains 97% overall accuracy and 96.6% balanced accuracy, with class–level recalls of 98% for AD, 94% for FTD and 97% for CN. Test-set performance remains equally strong, yielding a balanced accuracy of 97.1% and minimal off-diagonal errors. These results demonstrate that proposed hybrid-fusion further enhances EEGNetv4's ability to discriminate between dementia and control recordings, combining both average-power and temporal-dependency features into a compact, high-performing classifier.

#### 4.3.1 Comparison with other fusion strategies

Fusion techniques generally follow three traditional strategies i.e., early, intermediate, and late fusion. Early fusion stacks all channels first and sends them through a single encoder; intermediate fusion lets each modality form its own features before concatenation; late fusion keeps separate heads and averages their scores. In this study, all three baselines use the same EEGNetv4 backbone and identical training settings to ensure a fair comparison.

Table 10 presents results on test set and shows that early fusion delivers the strongest baseline, reaching 92.43% accuracy. Late fusion follows with 89.70% accuracy; it preserves strong AD performance (0.90 precision, 0.94 recall) but FTD recall drops to 0.82. Intermediate fusion performs worst, recording 80.30% accuracy and the lowest FTD recall (0.70), indicating that its feature-level combination fails to capture key complementary cues.

**Table 10:** Comparative analysis with traditional fusion techniques.

**Technique**	**Classes**	**Precision**	**Recall**	**F1-Score**	**Accuracy**
Early fusion	AD	0.94	0.93	0.94	92.43%
	FTD	0.88	0.91	0.90	
	CN	0.94	0.92	0.93	
Intermediate fusion	AD	0.85	0.94	0.89	80.30%
	FTD	0.75	0.70	0.73	
	CN	0.83	0.77	0.80	
Late fusion	AD	0.90	0.94	0.92	89.70%
	FTD	0.90	0.82	0.86	
	CN	0.92	0.94	0.93	

These findings show that the point at which signals merge strongly affects dementia detection performance. The hybrid fusion network, which first concatenates branch features and then averages three classifiers, raises both accuracy and balanced accuracy to 97.10%, surpassing every traditional strategy while introducing only a modest increase in parameters.

#### 4.3.2 K fold cross validation

**Figure 14:**
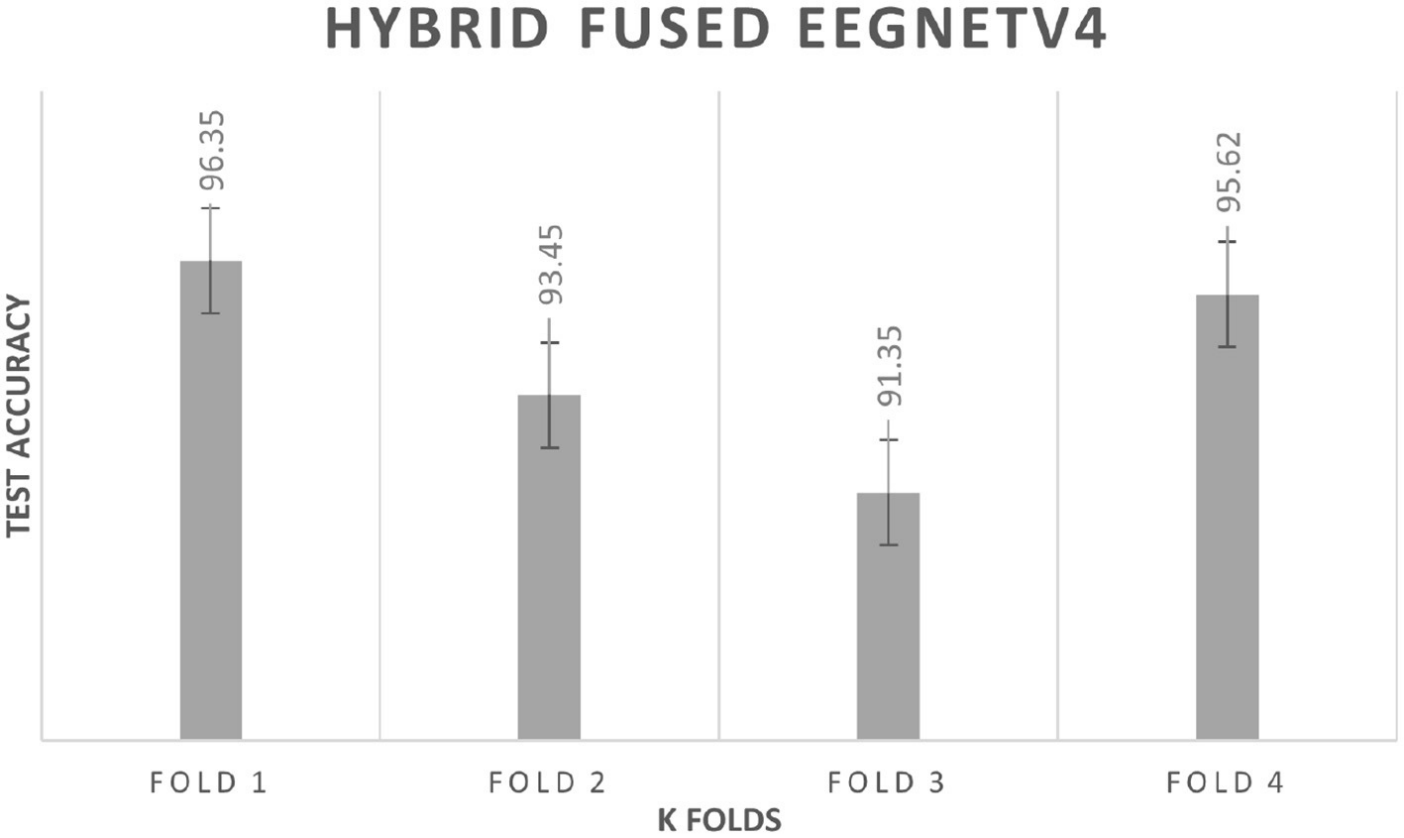
K fold cross validation results.

To rule out split-specific bias, a subject-wise 4-fold cross-validation is performed. Each fold keeps the original 80:20 ratio: 80% of the participants provide training windows, the remaining 20% test windows. Figure 14 plots the fold-wise test accuracy for the hybrid-fused EEGNetv4. Scores span 91.35% to 96.35% with a mean of 94.2% and a standard deviation of 2.3%. This narrow spread shows that the classifier stays robust across different subject partitions; the average accuracy is close to the 97.1% obtained on the independent test set, confirming that the model captures general EEG patterns rather than memorizing one data split.

### 4.4 Federated learning results

**Figure 15:**
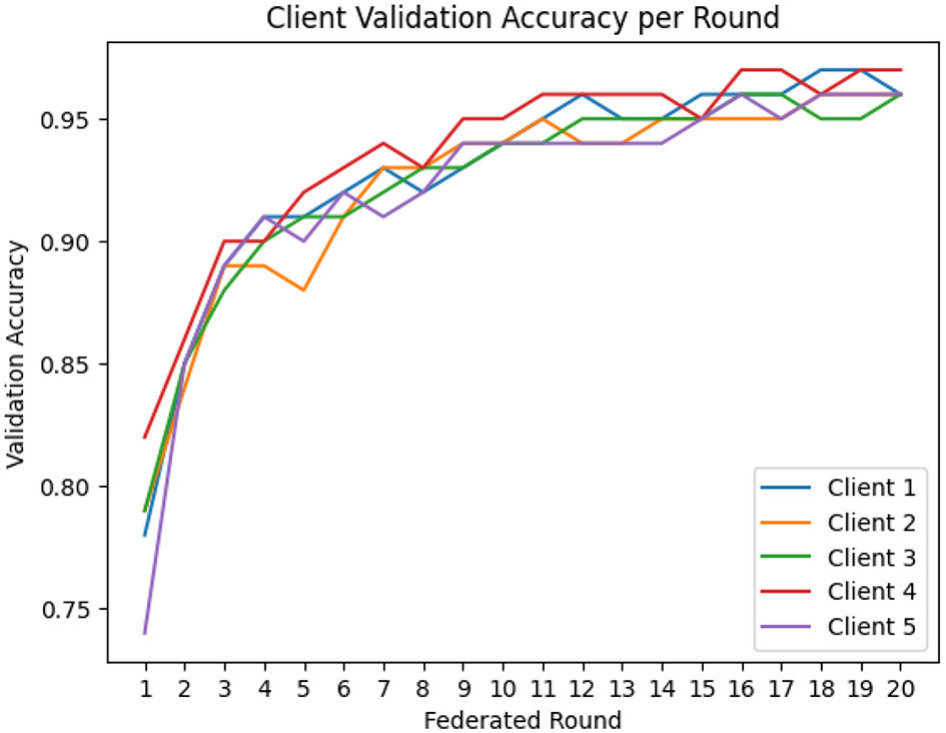
Client validation accuracy per federated round.

**Figure 16:**
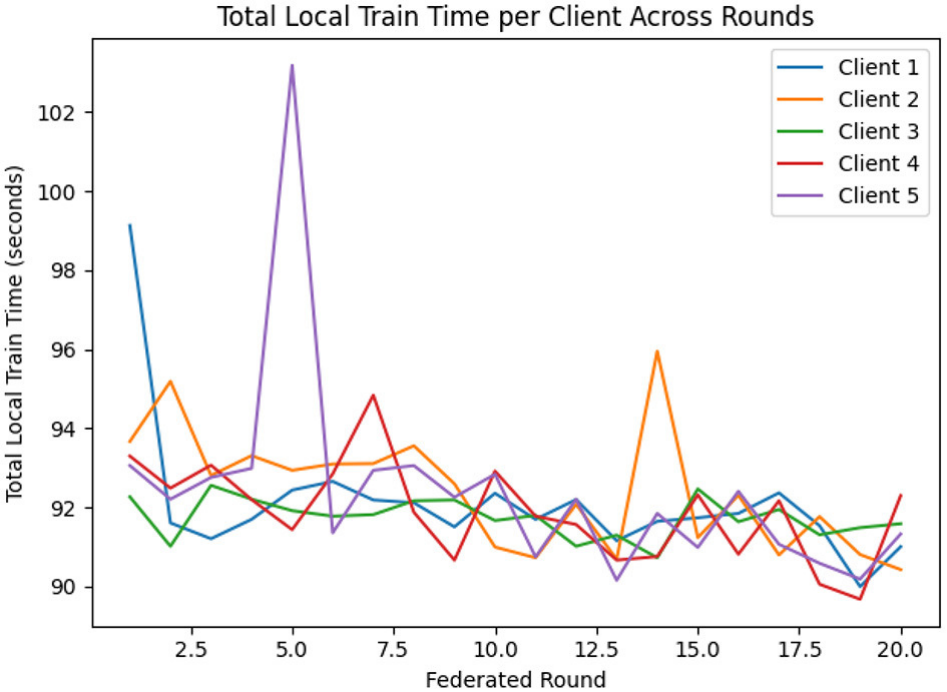
Total local training time per client across federated rounds.

For the implementation of FL, EEGNetv4 with proposed hybrid-fusion model has been utilized and trained for clients. Figure 15 shows the validation accuracy of each client over 20 federated learning rounds. All clients rapidly improve from an initial 0.78–0.82 in round 1 to above 0.90 by round 4, and converge between 0.95 and 0.97 by round 10. Figure 16 reports the total local training time per round: after a few initial fluctuations, all clients stabilize around 90–93 seconds per round, demonstrating efficient local updates.

Table 11 summarizes each client's final test-set performance after the last federated round. All five clients achieve an overall accuracy of at least 0.96, F1-scores of 0.96–0.98, and accuracies between 0.96 and 0.98. Client 5 records the lowest FTD recall (0.94), while clients 1–4 maintain recalls more than 0.97 for all classes.

**Table 11:** Clients test performance after federated training (with proposed hybrid–fusion EEGNetv4).

**Client**	**Accuracy**	**Precision**	**Recall**	**F1-score**
Client 1	0.97	0.97	0.97	0.97
Client 2	0.97	0.97	0.97	0.97
Client 3	0.98	0.98	0.98	0.98
Client 4	0.97	0.97	0.97	0.97
Client 5	0.96	0.96	0.96	0.96

**Figure 17:**
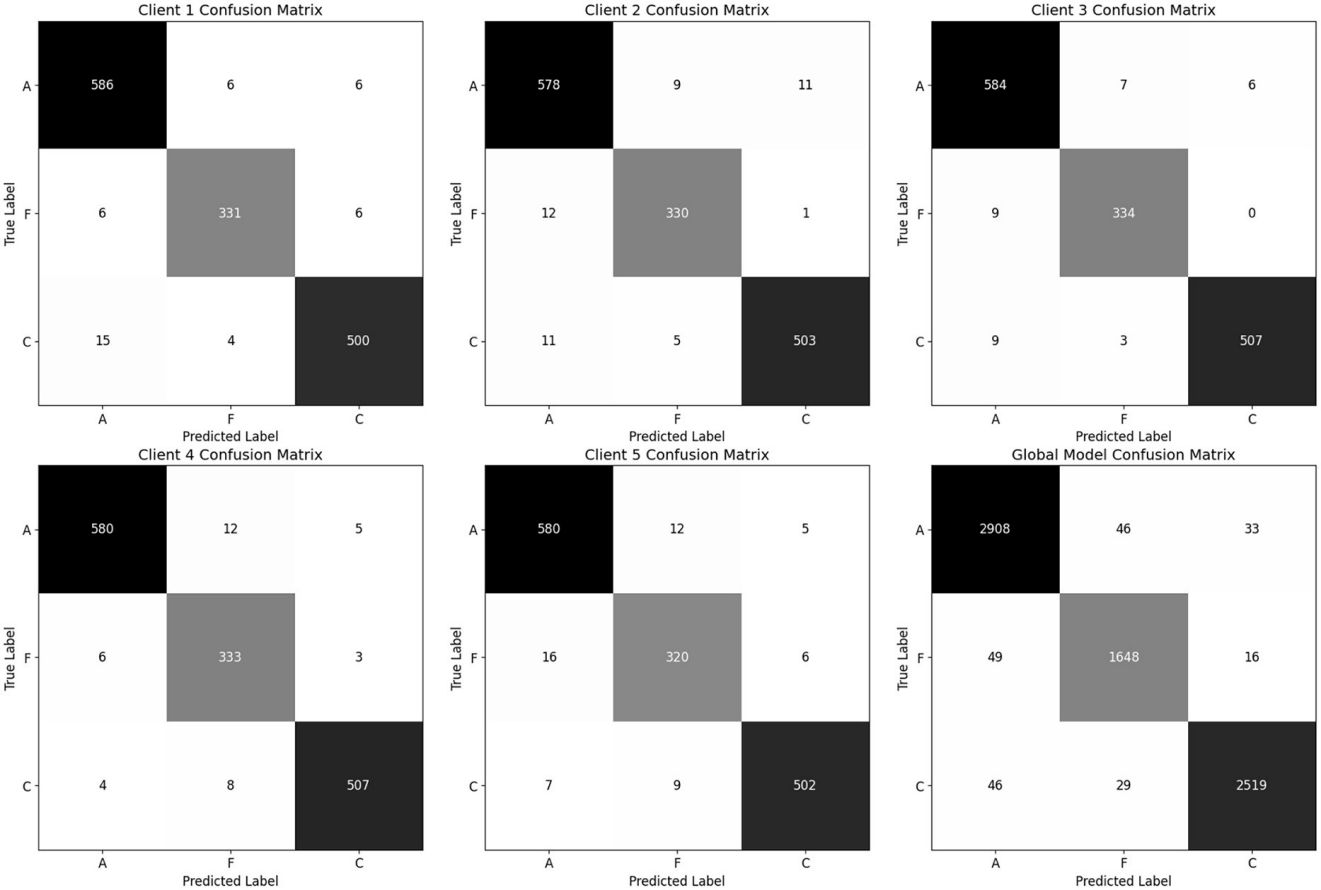
Confusion matrix for all clients and global model (aggregated model using Fed Avg) on test set.

The global model, obtained by averaging client updates (Equation 5), is evaluated on the test set (Table 12). It achieves 97% accuracy and a balanced accuracy of 6.9%, with per class recalls of 0.97 for AD, 0.96 for FTD and 0.97 for CN. The confusion matrix (as shown in Figure 17) on test confirms fewer than 50 misclassifications per class, underscoring the effectiveness of FedAvg in aggregating distributed knowledge without access to raw data.

**Table 12:** Global model test performance after federated training.

	**Precision**	**Recall**	**F1–score**	**Support**
AD	0.97	0.97	0.97	2,987
FTD	0.96	0.96	0.96	1,713
CN	0.98	0.97	0.98	2,594
Accuracy	97%			7,294
Balanced Acc.	96.9%			

Thus, the proposed hybrid–fusion EEGNetv4 model delivers consistently high performance across all clients and achieves marginally improved generalization as a global model, all while preserving data privacy.

### 4.5 Performance comparison with existing studies

Table 13 confirms that the hybrid-fusion EEGNetv4 outperformed on the public 88-subject EEG dataset. The nearest triple-class competitor, the coherence-based CNN of (Jiang et al., 2025), reports 94.3% accuracy, whereas the proposed model reaches 97.1%, a margin of roughly three percentage points. Other EEG studies either focus on easier binary contrasts such as AD vs. CN or AD vs. FTD (Zheng et al., 2025; Lal et al., 2024) or rely on handcrafted entropy or connectivity features, and none of them exceed 95% in the three-way setting. For Lakhan et al. (2024) achieves 99% accuracy, but the task is binary AD detection and the model fuses MRI, PET and EEG; the result is therefore not directly comparable to a pure-EEG, triple-class experiment. Even when the hybrid model trains in a privacy-preserving FedAvg setup it still delivers 97.0% accuracy. Thus, the dual-branch fusion network offers the highest reported accuracy for simultaneous AD, FTD and control discrimination using resting-state EEG, while remaining lightweight (1,609 parameters, less than 1 MB memory) and deployable in both centralized and federated environments.

**Table 13:** Performance comparisons with recent EEG dementia studies.

**Study**	**Model/features**	**Classification task**	**Accuracy**
Jiang et al. (2025)	Coherence-CNN	AD vs. FTD vs. CN	94.3 %
Zheng et al. (2025)	Functional-connectivity + SVM	AD vs. CN/FTD vs. CN	95 / 86 %
Lal et al. (2024)	SVD-entropy + KNN	Multiple binary pairs	91–93 %
Rostamikia et al. (2024)	Ranked EEG features + SVM	Dementia vs. CN/AD vs. FTD	93.5/87.8 %
Kassem and Kabbara (2024)	Graph neural network	AD vs. (FTD+CN)	92.33 %
Sahid et al. (2024)	Federated CNN	3-class	84.75 %
Lakhan et al. (2024)	FDCNN-AS (multimodal)	AD detection	99 %
Hybrid-fusion (central)	Dual-branch EEGNetv4	3-class	**97.10 %**
Hybrid-fusion (FedAvg)	Same model (federated)	3-class	**97.00 %**

The bold values represent the results of the proposed model presented in this paper.

## 5 Conclusion

This work demonstrates that DL models tailored for EEG signals can classify AD, FTD and CN from resting–state EEG. A publicly available 88 subject dataset comprising AD, FTD and CN cohorts was accessed. For data preprocessing band pass, notch filtering, exponential moving standardization, fixed length windowing and subject wise stratified splitting was utilized. For the DL models specific EEG tailored model was utilized e.g., EEGNetv1, EEGNetv4, EEGITNet, EEGInception, EEGInceptionERP. Among five evaluated architectures, EEGNetv4 achieved 96% test accuracy and, when extended with proposed hybrid-fusion, reaches 97.1% test accuracy using just 1,609 parameters. Furthermore, for decentralized training FL approach has been utilized with FedAvg method across five clients preserves privacy and yields global model with 96.9% accuracy, similar to the centralized results. These findings highlight that efficient the proposed hybrid-fusion and secure FL approach enable robust, resource–constrained deployment of EEG–based diagnostic tools in real–world clinical setting. However, the limitations included that FL setup (in practical) possess unbalanced data and with limited the client. Future work will focus on handling real client heterogeneity and designing more efficient FL strategies.

## Data Availability

The original contributions presented in the study are included in the article/supplementary material, further inquiries can be directed to the corresponding author. The datasets analyzed for this study can be found at DOI: 10.18112/openneuro.ds004504.v1.0.8.
